# ZnO Nanoparticles-Induced MRI Alterations to the Rat Olfactory Epithelium and Olfactory Bulb after Intranasal Instillation

**DOI:** 10.3390/toxics12100724

**Published:** 2024-10-05

**Authors:** Lifeng Gao, Yuguang Meng, Xiaowen Luo, Jiangyuan Chen, Xuxia Wang

**Affiliations:** 1 Department of Medical Imaging, School of Medicine, Jianghan University, Wuhan 430056, China; gao_lifeng@jhun.edu.cn (L.G.); aquariuswind@jhun.edu.cn (X.L.); chenjiangyuan@jhun.edu.cn (J.C.); 2 State Key Laboratory of Magnetic Resonance and Atomic and Molecular Physics, National Center for Magnetic Resonance in Wuhan, Key Laboratory of Magnetic Resonance in Biological Systems, Innovation Academy for Precision Measurement Science and Technology, Chinese Academy of Sciences, Wuhan 430071, China; mengyg@gmail.com

**Keywords:** zinc oxide, nanoparticles, toxicity, olfactory system, magnetic resonance imaging

## Abstract

Since zinc oxide (ZnO) nanoparticles (NPs) have been widely applied, the nano community and the general public have paid great attention to the toxicity of ZnO NPs. We detected 20-nm ZnO NPs biotoxicity following nasal exposure utilizing the non-invasive and real-time magnetic resonance imaging (MRI) technique. MR images were scanned in the rat olfactory epithelium (OE) and olfactory bulb (OB) on a 4.7 T scanner following the treatment (as early as 1 day and up to 21 days after), and the histological changes were evaluated. The influence of the size of the ZnO NPs and chemical components was also investigated. Our study revealed that 20-nm ZnO NPs induced obvious structural disruption and inflammation in the OE and OB at the acute stage. The results suggest that the real-time and non-invasive advantages of MRI allow it to observe and assess, directly and dynamically, the potential toxicity of long-term exposure to ZnO NPs in the olfactory system. These findings indicate the size-dependent toxicity of ZnO NPs with respect to the olfactory bulb. Further study is needed to reveal the mechanism behind ZnO NPs’ toxicity.

## 1. Introduction

Zinc oxide (ZnO) nanoparticles (NPs) have been employed in a wide range of rubber, cosmetic, foods, medical, and biological fields [1,2]. The potential health risks of mass-produced nanomaterials have received great public and academic attention. ZnO NPs’ aerosol exposure can originate from welding fumes and manufacturing processing [3,4], which has been proven to cause pulmonary impairment [5,6] and metal fume fever [7].

Studies have shown the translocation of nanosized particles from the nose to the central nervous system (CNS), an important mechanism by which these particles enter the brain. An early study demonstrated the anterograde axonal transport of 50-nm silver-coated gold colloids along the olfactory nerve to the olfactory bulb in the squirrel monkey after intranasal instillation [8]. Oberdörster, G. et. al. investigated the migration of inhaled ultrafine ^13^C particles (36 nm) in the rat brain and found that after 6 h of inhalation exposure, there was a significant and sustained increase in ^13^C concentration in the olfactory bulb, from which they inferred that the migration of ^13^C particles from the nasal cavity to the CNS was mainly accomplished via olfactory sensory neurons [9]. Likewise, metal oxide NPs such as manganese oxide (MnO) and iron oxide have been found to translocate into the brain via the olfactory route [10,11,12]. Therefore, we hypothesized that environmental and occupational exposures to ZnO NPs by the respiratory system, by virtue of their extremely small size, may enter the nasal cavity and affect the CNS via the olfactory pathway.

Nano-sized particles may exhibit distinct biological responses in contrast to bulk-sized particles of the same chemical composition [13]. Some cellular studies have reported the high cytotoxicity of ZnO NPs compared with larger-sized ZnO particles [14,15]. NPs were found to cause more immune function, inflammation, and transcriptomic responses than larger particles of the same material within the in vivo studies focused on nasal olfactory epithelium (OE), the respiratory tract, and oral exposure [16,17,18]. Tin-Tin-Win-Shwe et. al. demonstrated the size-dependent immune function of ultrafine carbon black in mice’s olfactory bulb (OB) following intranasal instillation [16]. After oral administration, ZnO NPs showed more transcriptomic responses than bulk-sized ZnO in rat liver [18]. Studies addressing the possibility of the effects of ZnO NPs versus larger-sized particles on the OB are very limited.

ZnO NPs have been manufactured in bulk and widely applied. The potential health risks of the mass-produced nanomaterials prompted us to pay attention to the NP’s safety application. ZnO NPs belong to highly soluble metal oxide. The results from cellular experiments demonstrate that ZnO NPs are more toxic than other metal oxide NPs [19,20,21]. Our understanding of how the ZnO NPs may disturb the olfactory pathway is poor. Cellular studies co-incubating human nasal mucosa cells with ZnO NPs illustrated cytotoxic, pro-inflammatory, and apoptosis responses in these cells [22,23]. Short-term inhalation exposure to ZnO NPs caused nasal necrosis in rats [24]. In the previous study, we proved that 30-nm ZnO NPs instilled in the nasal cavities of rats caused cellular injury and inflammation to the OE [25].

In this study, to further identify the influence of particle scale on the olfactory system (OE and OB), we applied magnetic resonance imaging (MRI) and histochemical methods to investigate the size effect of the intranasal exposure of nano-sized and submicron-sized ZnO particles (20 and 500 nm) on rats. Moreover, the influence of the chemical properties of the ZnO NPs and the zinc ions dissolved before the instillation was also considered.

The novelty of this article is that our study identifies the ZnO NPs-induced adverse effect on the olfactory bulb. As far as we know, olfactory bulb damage caused by ZnO NPs has never been reported before. The olfactory impairment caused by NPs could be screened by MRI. This work will facilitate the study of the link between ZnO NPs exposure and neurodegenerative disease.

## 2. Materials and Methods

### 2.1. Chemicals

ZnO NPs with a diameter of 20 nm and Fe_2_O_3_ NPs with a diameter of 30 nm were purchased from Haitai Nano Material Co., Ltd., Nanjing, China, and ZnO NPs with a diameter of 500 nm were purchased from National Pharmaceutical Chemical Reagent Co., Ltd., Beijing, China. We used sodium carboxymethyl cellulose (CMC) as an agent to suspend the NPs, which was manufactured by Sinopharm Chemical Reagent Co., Ltd., Shanghai, China. CMC is a water-soluble polymer that increases the viscosity of a solution, thereby helping to disperse nanoparticles and prevent aggregation. By forming a viscous solution, CMC effectively reduces the settling rate of the particles and therefore provides a good suspension aid.

### 2.2. Characterization of NPs

For the particle size characterization, the ZnO and Fe_2_O_3_ NPs were measured using JEM-200CX transmission electron microscopy (JEOL, Japan). The purity of the NPs was evaluated via an X-ray fluorescence spectrometer (Bruker, Germany). For the crystalline phase characterization, the NPs were analyzed via D/MAX 2000 X-ray diffraction (Rigaku, Japan). The specific surface area of the NPs was measured using an ASAP2010 Brunauer–Emmett–Teller technique. ZnO NP-CMC suspension was prepared by dispersing the ZnO NPs within 1 wt% CMC saline solution. The Fe_2_O_3_ NPs were also dispersed within the same solution to prepare Fe_2_O_3_ NP-CMC suspension. The concentration of Zn^2+^ in the supernatant of ZnO NP-CMC suspension was determined using complexometric titration: ammonia–ammonium chloride buffer solution (pH 10.0) was added to the suspension, followed by the addition of Chromium Black T indicator, and titration was carried out with a standard solution of 9.92 mM Na_2_EDTA. The endpoint of the titration was reached when the color of the mixture changed from sky blue to purplish red. The concentration of Zn^2+^ in the 20-nm and 500-nm ZnO NP-CMC suspensions was to be about 0.065 mg·mL^−1^ in both cases. The ZnO NP-CMC suspensions were centrifuged at 12,000× *g* rpm and/or 16,099× *g* for 10 min; then, the supernatants were extracted.

### 2.3. Animals

All experimental animals were handled in accordance with the National Committee for the Ethics and Use of Laboratory Animals. Male Sprague-Dawley rats (140–200 g in weight) were bought from the Animal Laboratory of Zhongnan Hospital of Wuhan University. The rats were group-housed in clean polypropylene cages in the SPF-level laboratory animal room. They are maintained within a light-cycle-controlled and temperature-controlled environment. Relative humidity was kept at 50 ± 5%. Rats are free to commercial rodent food and water. The animals were subjected to experimental studies following at least 5 days of laboratory domestication.

### 2.4. Experimental Preparation

Animals without obvious nasal structural abnormalities were chosen for intranasal exposure. Exposure doses were designed in the same way as our previous study [25]. Animals were randomly separated into five groups to receive unilateral intranasal drops of one of the following 40 μL of solution/suspension (given with a 20 µL pipette tip):

(1) 1 wt% saline solution of CMC, n = 8;

(2) Supernatant of 40 mg ZnO mL^−1^ 20-nm ZnO NP-CMC suspension (S-ZnO20), n = 5;

(3) 40 mg Fe_2_O_3_ mL^−1^ Fe_2_O_3_ NP-CMC suspension (Fe_2_O_3_), n = 8;

(4) 40 mg ZnO mL^−1^ 20-nm ZnO NP-CMC suspension (ZnO20), n = 8;

(5) 40 mg ZnO mL^−1^ 500-nm ZnO NP-CMC suspension (ZnO500), n = 8.

Before the intranasal exposure, all suspensions were sonicated for 10 min.

### 2.5. MRI Study

A Bruker Biospec 4.7 T/30 cm small animal scanner was utilized to perform all MRI scans. A 12 cm diameter Helmholtz volume coil was used for radiofrequency (RF) pulse transmission, and a 2.5 cm diameter single loop surface coil was used for signal reception, both of which were decoupled. The nasal structures of the animals were scanned via the spin-echo T_1_-weighted MRI sequence to avoid including unsuitable animals in the trials. At 0 days (before) and 1 day (1 d), 4 days (4 d), and 7 days (7 d) after intranasal instillation, T_2_-weighted image acquisition screening for OE defects and quantitative measurement of T_1_ values were performed on the OE of all rats under chloral hydrate anesthesia (5 wt% solution, 7 mL kg^−1^ dosage), respectively, to monitor changes in the OE dynamically in the following five groups: CMC (n = 6), S-ZnO20 (n = 5), Fe_2_O_3_ (n = 6), ZnO20 (n = 6), and ZnO500 (n = 6). At the same time points, T_2_-weighted images were acquired to monitor OB alterations in five groups (n = 3 in each group). At 21 days (21 d), the damage to the olfactory bulbs of the ZnO20 (n = 3) and ZnO500 (n = 3) groups were scanned with T_2_-weighted and T_1_ inversion recovery images. The spin-echo T_1_-weighted image parameters were as follows: repetition time: 400 ms; echo time: 15 ms; field of view: 1.5 cm × 1.5 cm; matrix size: 128 × 128; slice thickness: 0.8 mm; and number of averages: 2. The T_2_-weighted image parameters were as follows: a repetition time of 3000 ms; 6 echoes, with echo times ranging from 25 to 175 ms; and an echo interval of 25 ms. T_1_ values of the OE were measured by the fast Look–Locker T_1_ imaging measurement sequence (LL T_1_) with a repetition time of 5000 ms. The T_1_ values were fitted by 24 small-angle gradient echo signals acquired with an excitation interval of 150 ms. The imaging parameters for the T_1_ inversion recovery sequence were as follows: repetition time: 5000 ms; echo time: 15 ms; inversion recovery time: 450 ms. In the other MRI scans, the field of view parameters, matrix size, slice thickness, and number of averages are the same as those in the spin-echo T_1_-weighted image.

### 2.6. Histologic Examination

At 1 and 22 days after treatment, except for the S-ZnO20 group, two typical rats in every group were executed for pathohistological evaluation of the OE and OB. For hematoxylin–eosin staining, animals were perfused with 0.9 wt% NaCl and 4 wt% paraformaldehyde solutions through the left ventricular aorta. The OE and OB of each animal were then dissected, sampled, and fixed in 4 wt% paraformaldehyde solutions for one night. For the next 7 days, the fixed OE samples were decalcified within 15% EDTA. After that, the OE and OB specimens were embedded in paraffin and sliced to a thickness of 4 μm. The sections were deparaffinized, rehydrated, and stained with hematoxylin and eosin. At last, the sections were dehydrated, cleared, and covered with a neutral balsam.

### 2.7. Statistical Analysis

The region of interest (ROI) on the LL T_1_ image was selected in ectoturbinate 2 (susceptible region) of the instilled side of the OE in the CMC group, and the T_1_ value of ROI was fitted using a house-made MATLAB program. The data were expressed as mean ± standard deviation and statistically analyzed using the SPSS19.0 software package. Two-way analysis of variance (ANOVA) was used to analyze T_1_ value data. Two-tailed Student’s *t*-tests were used to evaluate the statistical significance of inter- and intro-group differences. The significance level was set at *p* < 0.05 with Bonferroni correction for multiple comparisons.

## 3. Results

### 3.1. Characterization Results of NPs

**Figure 1:**
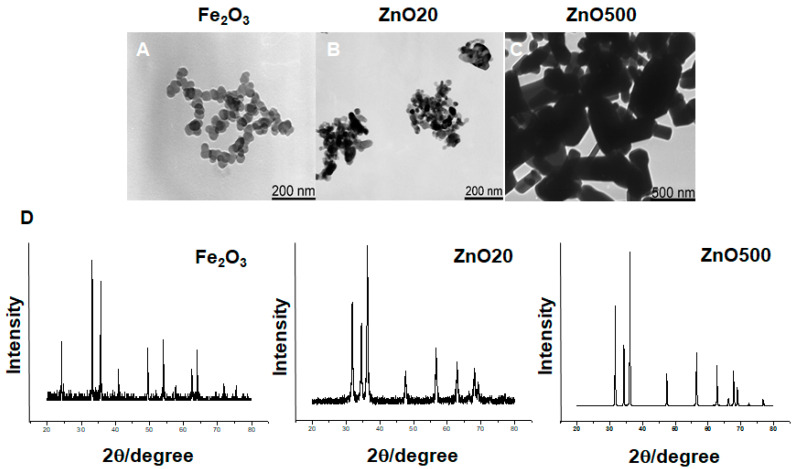
Representative transmission electron microscopy images and X-ray diffraction spectra of Fe_2_O_3_ nanoparticles (NPs), 20-nm zinc oxide (ZnO) NPs, and 500-nm ZnO NPs. (**A**–**C**) transmission electron microscopy images; (**D**) X-ray diffraction spectra. A and B: bar 200 nm; C: bar 500 nm. The TEM images and X-ray diffraction spectra of Fe_2_O_3_ NPs and 500-nm ZnO NPs are from the literature [25].

In Table 1, the range of the diameter of the ZnO NPs (20 nm) was between 15 and 30 nm, and the length range was between 20 and 40 nm. The purity was 99.9 wt%. The specific surface area was 31.5 m^2^⋅g^−1^. The crystalline structure was a zincite phase crystal. Figure 1B shows representative transmission electron microscopy images of 20-nm ZnO NPs. The characterization results of the Fe_2_O_3_ NPs and the ZnO NPs (500 nm) are shown in Table 1 and Figure 1.

### 3.2. Toxic Effects of 20-nm ZnO NPs

**Figure 2:**
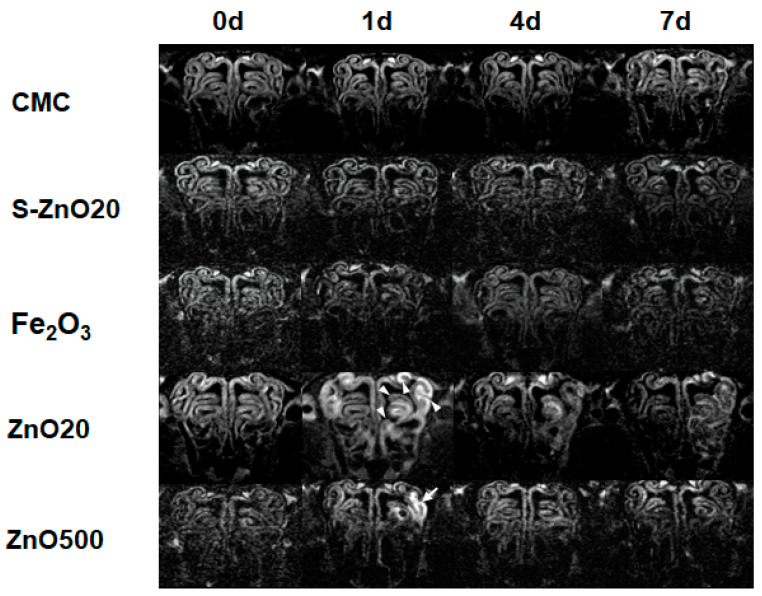
T_2_-weighted images of the OE before (0 d) and at 1, 4, and 7 days after exposure to CMC, S-ZnO20, Fe_2_O_3_, ZnO20, and ZnO500. The white arrow indicates the bright signal in the turbinate at 1 d in the ZnO20 group, while the white arrowhead marks the bright signal in the turbinate at 1 d in the ZnO500 group, suggesting different extents of edema.

T_2_-weighted images of the OE are shown in Figure 2. At 1 d, 4 d, and 7 d after intranasal exposure, the rats instilled with CMC, S-ZnO20, and Fe_2_O_3_ exhibited no evident abnormities in the OE compared to before the treatment (0 d). At 1 d, the rats treated with ZnO20 occasionally exhibited hyperintensity in areas of the OE in the bilateral turbinates, which can be explained by the septal window connecting two sides of the nasal cavity. The regions with hyperintensity were visible at 4 d and 7 d after the exposure to ZnO20. At 1 d, compared with the rats instilled with ZnO20, those treated with ZnO500 exhibited less-pronounced edema in OE. Edema was less apparent on T_2_-weighted images at 4 d and 7 d after the exposure to ZnO500.

**Figure 3:**
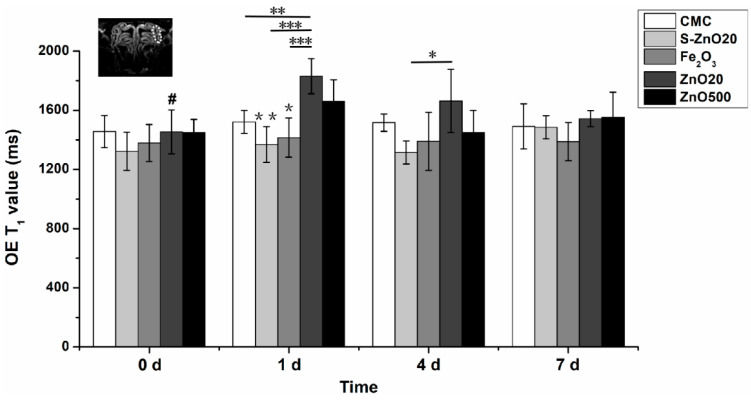
Histogram graph showing the T_1_ relaxation time (T_1_ value) of the region of interest (ROI) in the olfactory epithelium (OE) before (0 d) and after intranasal instillation in each group of rats. The white dashed line indicates the ROI in the ectoturbinate 2 of the OE in the CMC group. At 1 d, in the ZnO20 group, the T_1_ value of the ROI significantly increases compared with its baseline at 0 d (**#**, *p* = 0.003), which is significantly higher than that of the CMC, S-ZnO20, and Fe_2_O_3_ groups, respectively (**, *p* < 0.005, ***, *p* < 0.001). At 1 d, the T_1_ value in the ROI of the ZnO500 group is significantly higher than that of the Fe_2_O_3_ and S-ZnO groups, respectively (*, *p* < 0.05; **, *p* < 0.01). At 4 d, the T_1_ value of the ZnO20 group is significantly higher than that of the S-ZnO20 group (*, *p* < 0.05).

Figure 3 shows the T_1_ relaxation time (T_1_ value) changes in the ectoturbinate 2 of the instilled side of the OE in the CMC, S-ZnO20, Fe_2_O_3_, ZnO20, and ZnO500 group rats over time.

Two-way ANOVA revealed that the main effects of time, group, and time × group interaction were statistically significant for T_1_ value. At 1 d, the T_1_ value of the ZnO20 group significantly increased compared to its baseline (0 day) (1830.3 ± 118.5 ms vs. 1453.8 ± 148.6 ms, *p* = 0.003). It also significantly increased compared to the CMC group (vs. 1521.67 ± 78.2 ms, *p* < 0.005), S-ZnO20 group (vs. 1368.6 ± 121.3 ms, *p* < 0.001), and Fe_2_O_3_ group (vs. 1415.7 ± 132.4 ms, *p* < 0.001) at 1 d, respectively. There was a trend of a rise in the OE’s T_1_ value in the ZnO20 group relative to that in the ZnO500 group. After that, the T_1_ value of the ZnO20 group gradually decreased to 1664.3 ± 213.5 ms at 4 d and 1543.7 ± 54.7 ms at 7 d, respectively. At 4 d, the T_1_ value of the ZnO20 group was higher than that of the S-ZnO20 group (1664.3 ± 213.5 ms vs. 1314.8 ± 78.0 ms, *p* < 0.05). In the ZnO500 group at 1 d, a significant T_1_ value increase was seen compared to the Fe_2_O_3_ group (1661.2 ± 145.8 ms vs. 1415.7 ± 132.4 ms, *p* < 0.05) and S-ZnO20 (vs. 1368.6 ± 121.3 ms, *p* < 0.01), respectively. An upward trend was shown compared to its baseline (0 day) (vs. 1450.7 ± 88.2 ms). Then, the T_1_ value of the ZnO500 group returned to the normal level.

**Figure 4:**
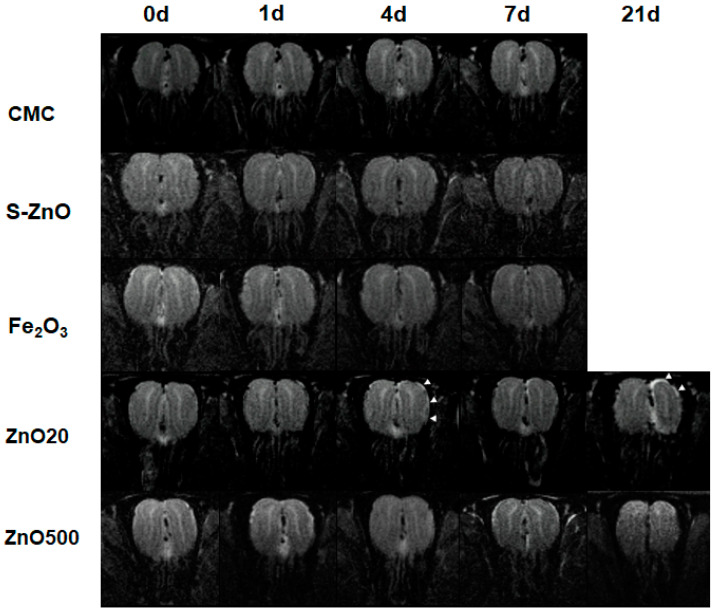
The T_2_-weighted image of the olfactory bulb (OB) before and after exposure to CMC, S-ZnO20, Fe_2_O_3_, ZnO20, and ZnO500. At 4 d and 21 d, the white arrow indicates the region of high signal intensity along the lateral and dorsal border of the right treated OB in the ZnO20 group, suggesting edema.

Figure 4 shows the T_2_-weighted images of the OB in one representative rat from the CMC, S-ZnO20, Fe_2_O_3_, ZnO20, and ZnO500 groups at each time point before and after intranasal instillation. At 4 d, bright signal intensity was shown in the ZnO20 group along part of the lateral and dorsal borders of the treated OB, which became more pronounced at 21 d. Transient bright signal intensity on the lateral border in the ZnO500 group was shown at 4 d. However, it returned to a normal level at 21 d. At 21 d, the treated OB size in the ZnO20 group showed a significant decrease compared with left untreated OB, while no apparent shrinkage was observed in the ZnO500 group. The treated OB in CMC, S-ZnO20, and Fe_2_O_3_ groups had not shown any changes in T_2_ signal intensity until 7 d.

**Figure 5:**
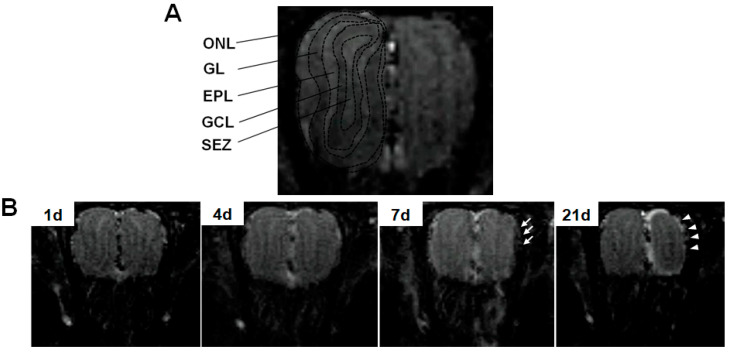
The inversion recovery T_1_-weighted images of the rat olfactory bulb (OB). In panel (**A**), the black dashed line outlines the distinct layer structure of the control side of the OB in the CMC group, from the outer layer to the inner layer, including the ONL (olfactory nerve layer), GL (glomerular layer), EPL (external plexiform layer), GCL (granule cell layer), and SEZ (subependymal zone). In panel (**B**), the inversion recovery T_1_-weighted images of the OB in the ZnO20 group at 1 d, 4 d, 7 d, and 21 d are shown. The arrow indicates the region of low signal intensity in the inversion recovery T_1_-weighted image on the instilled side, and the arrowhead reveals the thinning of the ONL.

Figure 5 shows the inversion recovery T_1_-weighted images of the rat OB at 1, 4, 7, and 21 days after intranasal instillation of ZnO20. At 7 d, there was an apparent region of low signal intensity along the lateral border of the instilled side of the OB, indicating tissue edema and inflammation. At 21 d, the instilled side of the OB significantly shrunk compared with the control side, probably due to the thinning of the olfactory nerve layer (ONL) to some extent.

### 3.3. Structural Changes of the OE and OB

**Figure 6:**
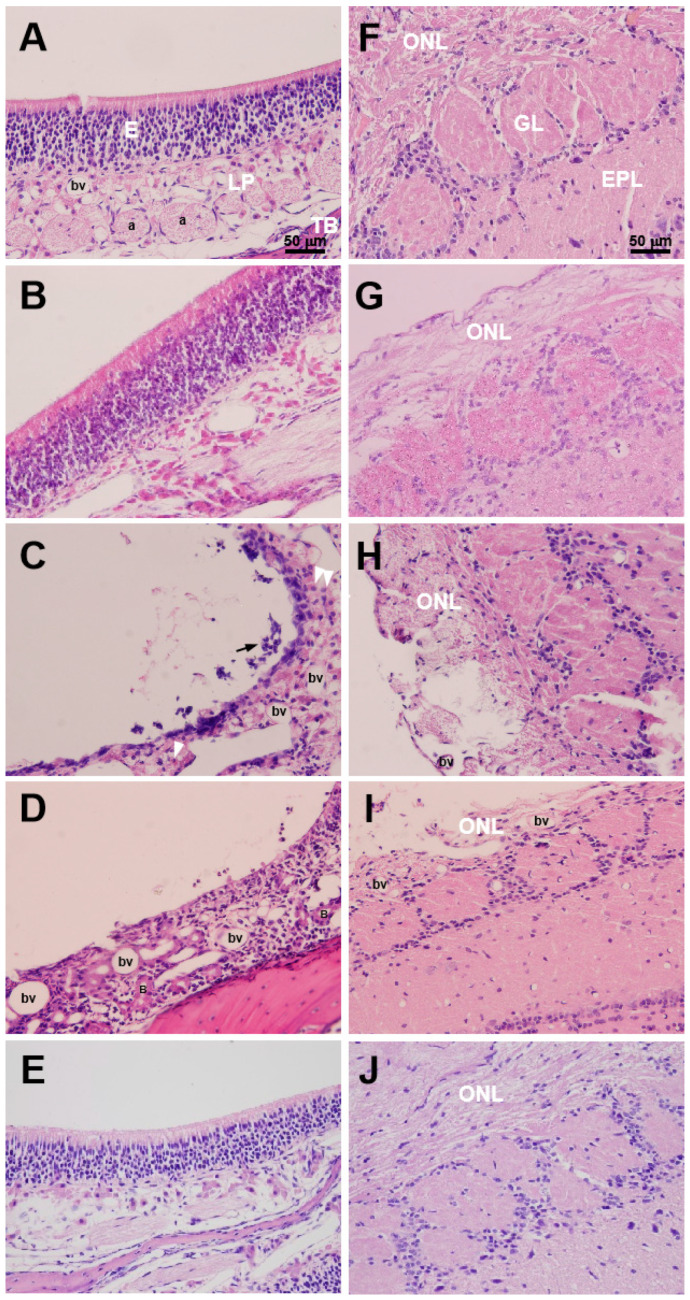
The hematoxylin–eosin staining images of rat olfactory epithelium (OE) and olfactory bulb (OB) after exposure to CMC, Fe_2_O_3_, ZnO20, and ZnO500. (**A**–**E**) represent the stainings of the OE at 1 d after exposure to CMC, at 22 d after exposure to Fe_2_O_3_, at 1 d and 22 d after exposure to ZnO20, and at 22 d after exposure to ZnO500, respectively. (**F**–**J**) represent the correspondent stainings of OB. The white arrowheads mark infiltrated phagocyte cells, and the black arrow points out the cellular debris in (**C**). TB: turbinate; LP: lamina propria; E: epithelial cells; a: bundles of olfactory axons; bv: blood vessel; ONL: olfactory nerve layer; GL: glomerular layer; EPL: external plexiform layer (bar: 50 µm).

In Figure 6, cellular damage and inflammation in OE, as well as olfactory nerve fiber losses in OB, are induced by 20-nm ZnO NPs. The normal structure of the OE in the CMC-treated rat is presented in Figure 6A, which is composed of epithelial cells (E), lamina propria (LP), and turbinate (TB). The olfactory axons penetrate and terminate in the olfactory glomeruli after traveling across the surface of the OB, which constitute the ONL (Figure 6F). The ZnO20 group showed an acute inflammatory cell infiltration in the OE at 1 d, with disorganized epithelial cells, accompanied by the disappearance or reduction of olfactory axons. At 22 d, it was likely an epithelial cell reproduction originating from the LP, indicating a recovery state of the injured OE. A vacuole was apparent in the ONL on the lateral OB as early as 1 day after the treatment. By 22 days after the treatment, the olfactory axons no longer formed an intact layer, and the thickness of the ONL was thinner than before. The blood vessels in the OE and OB of the ZnO20-treated rats were significantly dilated, suggesting increased vascular permeability due to inflammatory reactions. At 22 d, the hematoxylin–eosin staining results of the OE and OB in both the Fe_2_O_3_ and ZnO500 groups illustrated well-organized columnar epithelial cells and the compact structure of the ONL.

## 4. Discussion

The main findings of this study are that the qualitative and quantitative analysis on the OE and OB in rats using MRI and combined with histopathological examination confirm that intranasally instilled 20-nm ZnO NPs acutely results in cellular damage and inflammation in the OE, accompanied with edema and atrophy in the OB.

Our experimental results are consistent with the viewpoint of particle size as a major factor influencing the toxic effects of nanomaterials. The size of the NPs makes their movement in the body across cell membranes and normal diffusion barriers possible. We found that 20-nm ZnO NPs caused the most severe damage to the rat OE and OB. Quantitative T_1_ value measurement illustrated that at 1 d, the T_1_ value of the OE in the ZnO20 group increased compared to those in all of the other groups at the same time point, which was consistent with the T_2_-weighted imaging results, suggesting most severe edema [26] in the OE of the ZnO20 group. An obvious low signal intensity was observed in the inversion recovery T_1_-weighted image on the lateral side of the OB of the ZnO20 group at 7 d, which may mark tissue edema, followed by atrophy at 21 d. The MRI findings were identified via the histological results. Typical pathological features of the OE in the ZnO20 group were structural disorganization of the epithelial cell layer, disappearance or shrinkage of the olfactory axons, and infiltration of inflammatory cells; the OB was characterized by the thinning of the ONL. Changes in vascular permeability induced by inflammatory responses were present in both the OE and OB. For the 500-nm ZnO NPs, the instilled side of the OE and OB did not appear to be very obviously damaged, either from the MRI findings (only a transient olfactory epithelial edema highlight signal was visible on the T_2_ weighted image) or from the hematoxylin–eosin pathology findings. These results suggest that 20-nm ZnO NPs exert more toxicity responses to the OE and OB than those induced by 500-nm ZnO NPs. The olfactory nerve axon diameter is <200 nm [27], which may be the “threshold” limiting the uptake of particulate matter into the olfactory nerve terminals. Elder et al. [10] found that inhaled 30-nm MnO NPs could enter the OB via the olfactory nerve. Then, there were increases in Mn concentrations in the OB, striatum, frontal cortex, and cerebellum. Tumor necrosis factor-α protein, macrophage inflammatory protein-2, and glial fibrillary acidic protein were also increased in the OB. Bermudez et al. [28,29] found that 20-nm TiO_2_ NPs induced inflammatory responses and cytotoxicity in rats at a rate 5–10 times higher than 300-nm TiO_2_ NPs. Our results also found that the smaller the particle size, the more likely they were to cause injury to the OE and OB. Wang et al. [11] reported the TEM results of neurodendron degeneration, membranous structure disruption, and the lysosome increase in the OB after long-term and low-dose intranasal exposure to nano-sized Fe_2_O_3_ (21 nm). However, in our study, the MRI results at each time point did not show any signal abnormality in the OE and OB after Fe_2_O_3_ NPs exposure, and the hematoxylin–eosin pathology results did not show any significant damage (except for vasodilatation). Therefore, we conclude that Fe_2_O_3_ NPs did not cause obvious damage to the olfactory system of the rats in the present experiment. Fe_2_O_3_ NP was designed as a negative control in our study because it is classified as insoluble metal oxide. The element-dependent nanotoxicity of metal oxide NPs implies that the Zn element might be the toxicant form. Zn^2+^ could inhibit cellular energy production by blocking mitochondrial respiration and could even induce neuronal death. Given that ZnO NP is a kind of highly soluble metal oxide, we infer that the releases of zinc ions will rise as the particle size decreases. We suggest that the dissolution should be regarded as a crucial step in producing the toxicity of ZnO NPs.

There are some arguments about the induction of the adverse effects of ZnO NPs. Due to the high solubility of ZnO NPs, some in vitro studies have suggested that ZnO-NP cytotoxicity stems mainly from the release of Zn^2+^ ions [24,30,31]. However, several studies suggest that particle dependence is another important toxicity mechanism for ZnO NPs [32,33,34,35]. A review discussed that ZnO NP-induced genotoxicity could originate from the release of Zn^2+^ ions and particle form [36]. Given that the smaller the particles of substances with the same chemical properties are, the greater solubility they have, the [Zn^2+^] in both the supernatants of the 20-nm and 500-nm ZnO NP-CMC suspensions was measured in our study. Exceeding our expectations, the [Zn^2+^] is equal in both suspensions. Furthermore, the [Zn^2+^] in the supernatant of the 20-nm ZnO NP-CMC suspension did not induce significant defects to the OE and OB. We show that the dissolved fraction of the ZnO NPs in suspension does not account for the toxicity of the ZnO NP-CMC suspensions to the rat olfactory system, which is consistent with the results of our previous research [25] and the toxicity of the ZnO NP suspensions to Daphnia magna [33].

ZnO NPs are among the more soluble metal oxide nanomaterials. Therefore, it is more likely to be transformed into soluble zinc ions after in vivo treatment. Except for the particle size factor, dissolved Zn^2+^, as the other negligible aspect, may play a major role in the ZnO NPs’ toxicity to nasal mucosal cells [22]. Previous studies have found that intranasal instilled ZnSO_4_ can destroy OE, leading to peripheral afferent nerve deafferentation [37]. Persson et al. reported that Zn^2+^ (as ZnCl_2_) could be translocated along the olfactory nerves of the rat into the olfactory bulb and even the anterior portion of the olfactory cortex, and that zinc ions have been found to accumulate in the olfactory glomerulus axon terminals of primary olfactory neurons [38]. We speculate that the slightly acidic environment (pH 5.5–6.5) of the nasal mucosa [39] and the low pH within cytoplasmic vesicles (e.g., lysosomes, pH 5.2) facilitate the dissolution of ZnO NPs [40]. In line with this opinion, surface coatings on the ZnO nanoparticles mitigated cellular responses such as cell stress, inflammatory, and apoptosis [23]. Interestingly, accumulated Zn in the form of ZnO in rat liver has recently been revealed, demonstrating some overlaps and considerable specificity in metabolism profiles related to the antioxidant systems and energy metabolism pathways versus ZnSO_4_ exposure [35]. ZnO in particulate form in the OB synaptosomes and brain has been found following nasal exposure of rats to airborne ZnO NPs (12–14 nm particle size), demonstrating an OB–brain translocation pathway for ZnO NPs [41]. However, the brain-region-specific distribution of exogenous ZnO NPs was not further clarified in that study. In a sense, our study located the ZnO NPs-induced adverse effect in OB.

In clinics, olfactory impairment and deficits have been reported in neurodegenerative disorders such as Parkinson’s disease (PD) and Alzheimer’s disease (AD) [42,43]. Damage to the olfactory pathway may be an early sign of AD and PD [44,45]. Epidemiologic investigations have shown a link between excessive zinc exposure and demyelinating diseases [46]. Zinc dysregulation will lead to an increase in extracellular Zn^2+^ concentration, which may precipitate β amylase and play a role in Alzheimer’s disease [47]. Van Denderen et al. [48] suggested that a decrease in the metabolic activity in the olfactory brain would result in a drop of the nerve fiber density in the anterior cerebral artery after ZnSO_4_-induced loss of olfaction. A decrease in nerve fiber density in the precommunicating part of the anterior cerebral artery was also found in patients with Alzheimer’s disease [49]. Moreover, ZnO NPs have been reported to be involved in the pathogenesis of neuronal diseases [50] and cause neural stem cell apoptosis [51]. For occupational workers chronically exposed to zinc-containing powders, fumes from zinc-plating factories, and welding environments, ZnO NPs might deposit in the nasal mucosa and subsequently undergo cellular uptake of primary olfactory sensory neurons, enter the olfactory bulb via axonal transport, and arrive at the olfactory cortex or even deeper brain regions via the axonal transmission of secondary olfactory neurons, resulting in excessive Zn element overload in the brain. Suppose that inhalation of ZnO NPs adversely affects the olfactory system and induces or aggravates the development of neurodegenerative disorders; in this case, the health of the occupationally exposed population should be given more attention.

As a well-established technique in medical and biological applications, the value of MRI has been greatly underestimated for nanotoxicology studies. The results of this study indicate that MRI could be used as a bio-screening tool to assess the reverse consequences of nasal exposure to ZnO NPs. It is important to ensure the safe application of nanomaterials [52] by elucidating the biological effects of nanomaterials and then exploring ways to eliminate and avoid their nanotoxicity.

## 5. Conclusions

The global rise in nanomaterials production has prompted people to pay attention to the safe application of NPs. We found acute toxicity effects on the rat olfactory system using MRI technology via nasally instilled 20-nm ZnO NPs. Our study revealed that 20-nm ZnO NPs caused obvious structural disruption and inflammation to the OE and OB at the acute stage. The results of our study support the viewpoint of the size-dependent toxic effect of NPs. The T_1_ value of the OE in the ZnO20 group increased at 1 d, consistent with the T_2_-weighted imaging results, suggesting severe edema. OB edema and atrophy were illustrated in the inversion recovery T_1_-weighted images following cellular damage and inflammation in the OE. The MRI findings were identified via the histological results. Structural disorganization of the epithelial cell layer, shrinkage of the olfactory axons, and infiltration of inflammatory cells are typical pathological features in the OE; the OB was characterized by the thinning of the ONL. Changes in vascular permeability were present in both the OE and OB. To the best of our knowledge, OB damage caused by ZnO NPs has never been reported before. The results suggest that the real-time and non-invasive advantages of MRI allow it to directly and dynamically observe and assess the potential toxicity of long-term exposure to ZnO NPs in the olfactory system. More studies are required to pinpoint the mechanism behind the ZnO NPs’ toxicity.

In the future, our research will focus on the biosafety assessment of manufactured nanoparticles, including, but not limited to, the nanoparticle toxicity of long-term nasal exposure and the transport mechanism of nanoparticles in neurodegenerative disease.

## Figures and Tables

**Table 1:** The characterization results of Fe_2_O_3_ NPs, 20-nm and 500-nm ZnO NPs (the data of Fe_2_O_3_ and 500-nm ZnO NPs are from the literature [25]).

Samples	Purity (%)	Diameter (nm)	Specific Surface Area (m^2^⋅g^−1^)	Crystalline Structure
Fe_2_O_3_	97.9	25–40	10.0	Maghemite
ZnO20	99.9	Width = 15–30 Length = 20–40	31.5	Zincite
ZnO500	>99.9	Width = 240–440 Length = 360–660	51.1	Zincite

## Data Availability

The data are contained within the article.
